# Isolated Unilateral Heptadactyly With Combined Preaxial and Postaxial Polydactyly of the Foot in a 9‐Month‐Old Infant

**DOI:** 10.1155/cro/3435692

**Published:** 2026-06-18

**Authors:** Benardine Phillip Mallilah, Emmanuel Pastory Marua, Cuthbert Beita Kafanabo, Getrude Laurent Mwasulile, Philipo Godliving Moshi

**Affiliations:** ^1^ Department of Orthopedics, St. Joseph Designated Hospital, Moshi, Kilimanjaro, Tanzania; ^2^ School of Medicine, KCMC University, Moshi, Kilimanjaro, Tanzania, kcmuco.ac.tz

**Keywords:** congenital foot anomaly, foot polydactyly, heptadactyly, postaxial polydactyly, preaxial polydactyly

## Abstract

**Background:**

Polydactyly is a common congenital limb anomaly characterized by supernumerary digits, most frequently involving the hands or feet. Foot polydactyly is traditionally classified into preaxial, postaxial, and central types, with postaxial forms being the most common and central forms the rarest. Heptadactyly, defined as the presence of seven digits in a single limb, represents an exceptionally rare and severe expression within this spectrum, most often described as central duplication. Isolated unilateral heptadactyly of the foot with combined preaxial and postaxial duplication is exceedingly rare, with very few cases reported in the literature.

**Case Presentation:**

We report the case of a 9‐month‐old female infant who presented with a congenital deformity of the right foot noted at birth. Clinical examination revealed unilateral heptadactyly with seven well‐formed toes involving both preaxial and postaxial duplication, resulting in medial and lateral widening of the forefoot. The contralateral foot, upper limbs, and systemic examination were normal, and there was no family history of congenital anomalies. Radiographic evaluation confirmed complete duplication of the medial and lateral rays without central ray involvement, tarsal abnormalities, or syndactyly, consistent with isolated mixed‐pattern preaxial–postaxial heptadactyly. Given concerns regarding footwear and future gait function, elective surgical correction was performed at 9 months of age. The procedure involved excision of the most medial and lateral supernumerary rays with meticulous preservation of neurovascular structures and reconstruction of soft tissues to restore a narrowed, stable, plantigrade foot. No internal fixation or ligament reconstruction was required due to preserved postoperative stability. The postoperative course was uneventful, with satisfactory wound healing, improved cosmetic appearance, and symmetric early functional use of the limb.

**Conclusion:**

This case highlights an exceptionally rare presentation of isolated unilateral heptadactyly with combined preaxial and postaxial duplication. It expands the phenotypic spectrum of foot polydactyly and emphasizes the importance of detailed clinical and radiological assessment for accurate classification and surgical planning. Early individualized surgical management can achieve favorable functional and cosmetic outcomes, while long‐term follow‐up remains essential to monitor gait development and detect late deformities.

## 1. Introduction and Background

Polydactyly is a congenital limb anomaly characterized by the presence of supernumerary digits affecting the hands and/or feet [1]. It represents one of the most common congenital limb malformations, with an estimated occurrence ranging from 0.3 to 3.6 per 1000 live births and up to 10.7 per 1000 in certain populations. The condition demonstrates ethnic variability, with higher prevalence reported in African populations, and a male predominance of approximately 2:1 has been described in several epidemiological studies [2, 3]. Importantly, polydactyly is extremely heterogeneous phenotypically, with substantial variation in anatomical configuration, severity, and associated anomalies across individuals and populations [1, 4].

Polydactyly of the foot is commonly classified using the Temtamy and McKusick framework into preaxial, postaxial, and central types. Preaxial polydactyly involves duplication of the medial ray, typically affecting the hallux; postaxial polydactyly affects the lateral ray involving the fifth toe; and central polydactyly involves duplication of the intermediate rays [5, 6]. Postaxial variants are the most common, whereas central duplication is distinctly rare. Mixed‐pattern duplication, involving both medial and lateral components or complex multiray configurations, represents a less frequently encountered entity and often presents unique reconstructive challenges [4, 7].

Heptadactyly, defined as the presence of seven digits in a single limb, is a rare and severe manifestation within the spectrum of polydactyly. Most reported cases of pedal heptadactyly involve duplication of central rays, occasionally producing seven fully formed digits with corresponding metatarsals. Classic reports describe isolated central heptadactyly with complete digital rays and no tarsal abnormalities, whereas other cases have been associated with complex limb malformations such as tibial aplasia. Broader reviews of complex polydactyly patterns consistently identify heptadactyly as an uncommon presentation, particularly when occurring in isolation [8–10].

Polydactyly may occur as an isolated anomaly or as part of a syndromic spectrum. Numerous genetic and developmental conditions have been associated with polydactyly, including syndromes involving cardiac, vertebral, renal, and skeletal abnormalities. These associations are particularly emphasized in complex and preaxial forms. Consequently, a comprehensive clinical and systemic evaluation is recommended in all patients presenting with multiple ray duplications to exclude additional anomalies and guide management [4, 6, 7]. In addition, polydactyly has been reported more frequently in the left foot than the right, underscoring the importance of documenting laterality in clinical reports [11].

Although isolated preaxial, postaxial, and central polydactyly have been widely described, mixed‐pattern duplications combining medial and lateral rays remain uncommon. Reports describing heptadactyly with combined preaxial and postaxial duplication are particularly rare and are more often encountered in syndromic or multilimb involvement such as tetrapolydactyly. Isolated unilateral heptadactyly of the foot with both preaxial and postaxial components in an otherwise healthy infant remains sparsely documented in the literature [8–10].

Within this context of marked phenotypic heterogeneity and the rarity of mixed pattern heptadactyly, the present case describes an infant with isolated unilateral heptadactyly of the foot involving both preaxial and postaxial duplication. This report highlights the anatomical configuration, radiological classification, surgical strategy, and early clinical outcome, contributing to the limited literature on complex mixed‐pattern polydactyly of the foot.

This case report was prepared in accordance with the CARE (CAse REport) reporting guidelines, and a completed CARE checklist is provided as Supporting Information [Available here].

## 2. Case Presentation

A 9‐month‐old female infant was brought by her mother with a congenital anomaly of the right foot, noticed at birth. The mother reported the presence of additional toes on the right foot that had not changed in size or appearance since delivery. There was no history of pain, ulceration, infection, or limitation in limb movement. The primary concerns were cosmetic appearance and potential future functional impairment, particularly regarding footwear and gait.

The child was born at a gestational age of 39 weeks and 6 days via spontaneous vaginal delivery to a 28‐year‐old Gravida 2 Para 1 mother. The antenatal period was uneventful, with regular antenatal clinic attendance and adherence to routine supplements. The mother denied any chronic illness, infections, teratogenic drug use, smoking, alcohol consumption, or radiation exposure during pregnancy. She remained normotensive and normoglycemic throughout gestation, and no obstetric complications were recorded during labor or delivery.

The infant was the second child born to nonconsanguineous parents. There was no family history of polydactyly, other limb anomalies, or known genetic disorders on either the maternal or paternal side. The firstborn sibling is healthy with no congenital abnormalities. This pattern is consistent with the frequent, sporadic occurrence of polydactyly in the absence of a family history, despite its often autosomal‐dominant inheritance.

### 2.1. Clinical Examination

**Figure 1:**
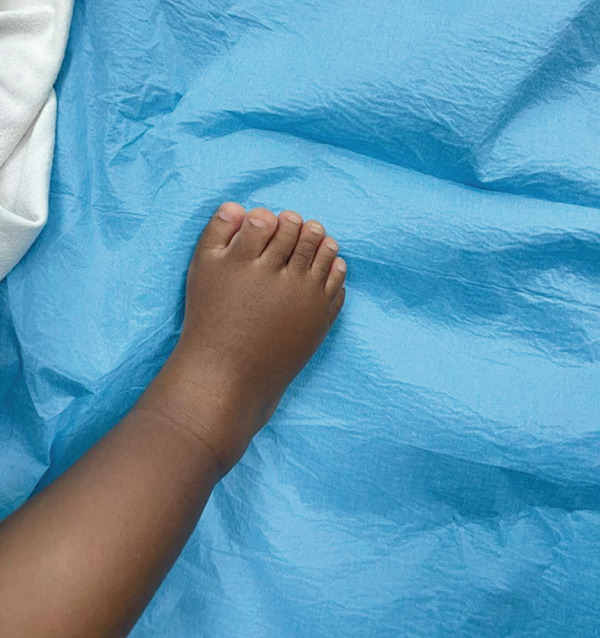
Clinical photograph of the right foot demonstrating unilateral heptadactyly with seven toes. Both preaxial (medial) and postaxial (lateral) duplication are present, resulting in widening of the forefoot.

On presentation, the infant was in good general condition, with normal vital signs and growth parameters appropriate for age. There were no dysmorphic facial features. Systemic examinations, including cardiovascular, respiratory, abdominal, and neurologic systems, were unremarkable; based on the absence of clinical indicators, further imaging including echocardiography and abdominal ultrasound was not indicated. No other congenital anomalies were detected. Local examination of the right foot revealed heptadactyly, with a total of seven toes. Both preaxial and postaxial polydactyly were present: an additional medial toe adjacent to the great toe and a lateral extra toe adjacent to the fifth toe, resulting in medial and lateral widening of the forefoot. The supernumerary digits were well‐formed soft tissue digits, each with normal appearing skin and nails. There were no signs of inflammation, ulceration, callosity, or skin breakdown. Passive and spontaneous movements at the ankle and all toes were full and painless, with preserved gross neurovascular status (Figure 1).

The left foot displayed a normal configuration with five toes and no deformity. Both upper limbs were normal, with no polydactyly or syndactyly. There were no limb length discrepancies or gross malalignment of the lower extremities.

### 2.2. Diagnostic Assessment (Imaging)

**Figure 2:**
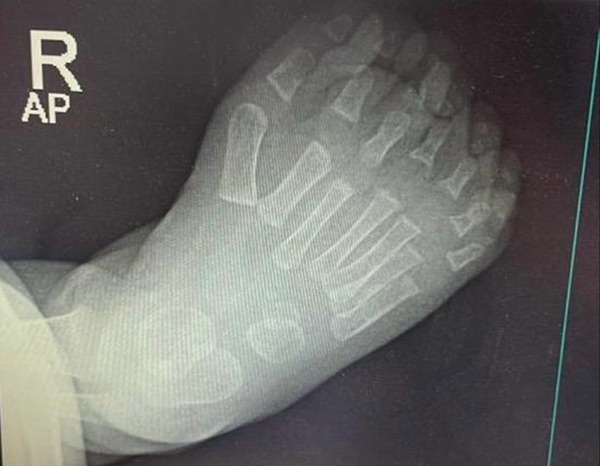
An anteroposterior radiograph of the right foot showing complete duplication of the medial and lateral rays with independent metatarsals and phalanges. No central ray duplication or tarsal abnormalities are identified.

**Figure 3:**
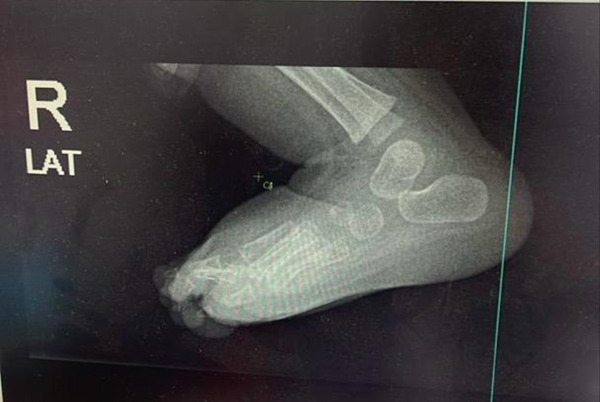
A lateral view of the plain radiograph of the right foot.

Radiographic evaluation demonstrated a duplicated medial ray with an accessory proximal phalanx and metatarsal, consistent with preaxial polydactyly, and a duplicated lateral ray with an accessory fifth metatarsal and additional phalanges, consistent with postaxial polydactyly (Figures 2 and 3). The duplicated rays were structurally independent, with no shared metatarsal bases or synostosis. There was no evidence of central ray duplication, tarsal bone anomalies, or longitudinal epiphyseal bracket (delta phalanx), and overall hindfoot and midfoot alignment were preserved. The central rays (second to fifth) were normally aligned and morphologically intact.

Based on these findings, the deformity is best classified within the Temtamy and McKusick classification as a mixed‐pattern polydactyly, demonstrating combined preaxial (medial) and postaxial (lateral) duplication within a single foot, without central involvement, thereby distinguishing it from central or mirror‐foot variants. Further subclassification indicates that the medial duplication corresponds to a metatarsal‐level first‐ray duplication according to the Watanabe classification, given the presence of an accessory hallux with its own metatarsal, although without duplication of the tarsal articulation, confirming it is not a true mirror duplication.

On the lateral side, the deformity represents a postaxial Type A polydactyly, characterized by a fully developed supernumerary sixth ray with well‐formed metatarsal and phalangeal elements. In radiographic–morphologic terms, this reflects a complete lateral ray duplication without metatarsal base sharing. Overall, these features support the diagnosis of isolated unilateral mixed preaxial–postaxial heptadactyly of the right foot, a complex and uncommon configuration that is not fully encompassed by traditional single‐axis classification systems and is best described as a hybrid subtype.

Radiological evaluation was based on structured morphological assessment of ray number, alignment, and metatarsal independence. Objective interpretation confirmed complete duplication of both medial and lateral rays with independent metatarsals and phalanges, without evidence of synostosis, tarsal involvement, or central ray duplication. Although formal angular measurements were not performed, qualitative classification was performed using established Temtamy–McKusick and Watanabe frameworks to ensure standardized anatomical characterization.

Diagnostic reasoning was based on the correlation of clinical and radiographic findings. Differential considerations included central polydactyly, mirror foot, and complex syndromic polydactyly. Central polydactyly was excluded because the central rays were preserved and normally aligned without duplication. Mirror foot was considered unlikely because there was no duplication of tarsal elements or complete medial ray repetition. The absence of associated congenital anomalies, normal systemic examination, and lack of syndromic features supported the diagnosis of isolated unilateral mixed preaxial–postaxial heptadactyly.

### 2.3. Therapeutic Intervention

In view of the caregivers′ concerns and the anticipated difficulties with footwear and gait due to the broadened forefoot, elective surgical correction was planned. The primary objectives were to achieve a plantigrade, stable, and pain‐free foot with five well‐aligned toes and an acceptable cosmetic appearance. Surgery was performed under general anesthesia at 9 months of age.

Preoperative planning involved a detailed clinical and radiographic assessment to determine optimal ray preservation and excision. The decision regarding ray excision was guided by alignment, anatomical development, and contribution to forefoot widening. The most medial and most lateral supernumerary rays were selected for removal, as both demonstrated unfavorable alignment and significantly contributed to increased transverse forefoot width, particularly in association with angulation of the first metatarsal.

The central rays (second to sixth digits) were preserved, as they exhibited well‐formed metatarsals and phalanges with satisfactory axial alignment, making them most suitable for maintaining a functional and shoe‐compatible foot.

Elliptical (racquet‐shaped) skin incisions were designed around the medial and lateral supernumerary digits to allow en bloc excision while preserving adequate soft tissue for contouring of the web spaces. Dissection was carried down to the level of the metatarsophalangeal joints and metatarsals. The supernumerary preaxial and postaxial digits were excised at their bases, including the associated phalanges and metatarsals. The excised rays were anatomically independent, with no shared joint structures or significant soft‐tissue interconnections with adjacent digits. Therefore, reconstruction of intermetatarsal ligaments or tendons was not required, as excision did not compromise transverse stability or alignment of the preserved rays. Neurovascular bundles supplying the accessory digits were carefully identified, ligated, and cauterized to ensure hemostasis and minimize the risk of neuroma formation, while those supplying preserved digits were protected.

Following excision, satisfactory alignment and spacing of the remaining rays were achieved without instability. Consequently, no K‐wire fixation was utilized, as there was no evidence of malrotation, joint instability, or risk of postoperative displacement that would necessitate internal stabilization. Similarly, periosteal balancing and corrective osteotomy were not required, as no residual angular deformity or soft‐tissue imbalance was observed intraoperatively. Soft tissue reconstruction was completed with meticulous layered closure and reshaping of the web spaces to recreate a normal toe pattern and narrow the forefoot. Hemostasis was secured, and a sterile dressing was applied.

Postoperatively, the limb was immobilized using a below‐knee posterior back slab, providing adequate protection and support during the initial healing phase. The procedure was completed without intraoperative complications, and the infant tolerated anesthesia well.

**Figure 4:**
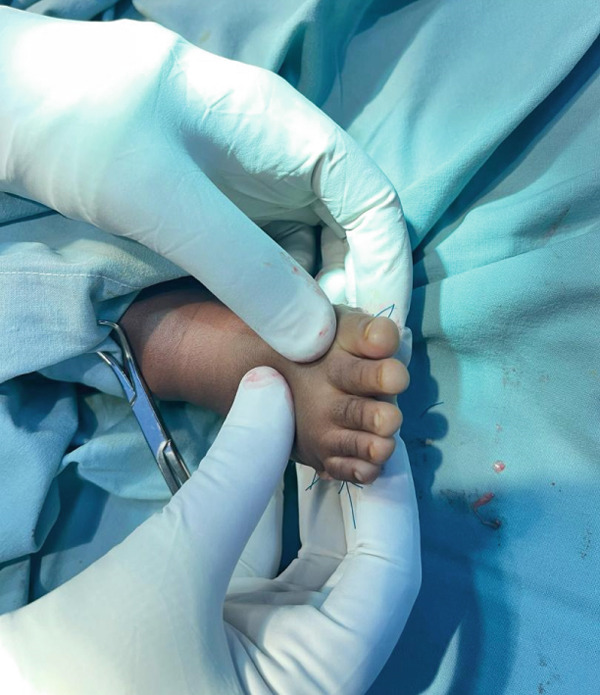
The well‐aligned right foot postoperatively.

Postoperatively, there was a clear reduction in transverse forefoot width with restoration of a five‐ray alignment and plantigrade foot positioning, in contrast to the preoperative broadened forefoot with seven‐digit configuration and medial–lateral widening (Figure 4). This anatomical correction was associated with improved early weight‐bearing symmetry and cosmetic appearance. A chronological summary of the patient′s clinical course is provided in Table 1.

**Table 1:** Clinical timeline.

Age/time point	Event
Birth	Right foot congenital anomaly with seven toes noted at delivery
Neonatal period	No associated symptoms, infections, ulceration, or functional impairment observed
9 months	Presentation at the orthopedic service for evaluation and radiographic assessment
9 months	Clinical examination and radiographic assessment confirmed a diagnosis of isolated unilateral mixed preaxial–postaxial heptadactyly.
9 months	Surgical planning and caregiver counseling were done, then excision of the medial and lateral supernumerary rays
Postoperative (Days 1–5)	Stable recovery without complications
2 weeks	Wound healing satisfactory
6 weeks	Preserved alignment and symmetric lower limb movement
3 months	Good cosmetic appearance, stable foot, age‐appropriate functional progression

### 2.4. Follow‐Up and Outcomes

The immediate postoperative period was uneventful. The wounds healed primarily, with no signs of infection, hematoma, or wound dehiscence. There was no clinical evidence of neurovascular compromise of the preserved toes.

On early follow‐up, the infant demonstrated symmetric spontaneous movement of both lower limbs, with full passive range of motion of the ankle and toes of the operated foot. The caregivers reported no discomfort, feeding difficulties, or changes in behavior attributable to pain. No follow‐up radiographs were obtained, as serial clinical evaluations demonstrated satisfactory alignment, stability, wound healing, and age‐appropriate functional progression, with no clinical indications necessitating repeat imaging.

At subsequent visits, as the child approached developmental milestones of standing and early supported ambulation, use of the right foot was symmetric with the Left. The forefoot appeared narrower, with five well‐aligned toes and a satisfactory cosmetic appearance. No areas of abnormal pressure, callosity, or skin breakdown were reported.

At the most recent review (3 months postprocedure), the child was meeting appropriate developmental milestones, and the caregivers reported no functional concerns or limitations. Long‐term follow‐up into later childhood is planned to monitor gait, footwear tolerance, and potential late deformities such as valgus deviation or residual forefoot widening. However, functional assessment remains limited due to preambulatory status. Validated gait scoring systems and pedobarographic analysis were not applicable at this stage.

### 2.5. Patient/Caregiver Perspective

The patient′s mother expressed satisfaction with the surgical outcome, particularly the improved appearance of the foot and the reduction in forefoot width. She reported no postoperative concerns regarding pain, wound healing, or limb function and was pleased that the child was achieving expected developmental milestones. The family remains committed to attending long‐term follow‐up visits to monitor future gait development and footwear tolerance.

## 3. Discussion

### 3.1. Rarity and Phenotype

Foot polydactyly spans from rudimentary soft‐tissue nubbins to complex multiray duplication, but heptadactyly of the foot remains exceptionally rare [7, 8, 12]. Isolated heptadactylia has been described mainly as central polydactyly with seven complete rays and no associated anomalies [8, 12]. Heptadactyly is also only sporadically mentioned within a broader series of complex polydactyly and mirror‐foot deformities, underscoring its rarity [7, 12, 13].

The present case differs by showing combined preaxial and postaxial duplication in a single foot, without central ray involvement, representing a mixed‐pattern heptadactyly that is far less frequently reported than pure central forms or mirror foot [8, 14, 15]. The absence of syndactyly, contralateral deformity, or systemic anomalies is consistent with isolated, nonsyndromic foot polydactyly but remains unusual for such a severe duplication pattern [7, 14, 15]. Previously reported cases of heptadactyly identified from the literature are summarized in Table 2.

**Table 2:** Published reports of heptadactyly and complex seven‐digit foot deformities identified in the literature.

Author (year, title)	Country	Age, Sex	Pattern	Syndromic/isolated	Side	Surgical plan	Treatment outcome
[8] *Isolated Heptadactylia*	Belgium	14 months, F	Central rays	Isolated	Left foot	Resection of second and third rays, IMT ligament reconstruction	Good functional and cosmetic outcome
[9] *A Rare Combination of Heptadactyl and Hexadactyl Polydactyly in a Neonate*	India	Neonate, M	Mixed (hepta + hexa)	Nonsyndromic	Bilateral	Excision of supernumerary digits	Good early outcome
[10] *Tetrapolydactyly: A Rare Presentation and Review of the Literature*	Japan	9 months, F	Complex (incl. heptadactyly)	Syndromic	Bilateral	Staged reconstruction	Functional improvement
[16] *Familial Crossed Polysyndactyly*	United States	NS, NS	Complex	Syndromic	Bilateral	NS	NS
[17] *An Unusual Case of Polydactyly of the Foot*	United States	60 years, M	Preaxial	Nonsyndromic	Right foot	NA	NA
[18] *A Seven Toes Foot: Case Series as an Isolated Dysplasia With Variety of Appearance*	Greece	5–24 months, M	Preaxial, central, and unspecified	Syndromic	Mixed	Removal of supernumerary rays	Good outcome from 1/2 to 9 years
Present case. 2025	Tanzania	9 months, F	Preaxial and postaxial	Nonsyndromic	Right foot	Resection of the medial and lateral rays	Good outcome for a 3‐month follow‐up

*Note:* To our knowledge, this represents one of the very few reported cases of isolated mixed preaxial–postaxial heptadactyly and may be the first such case reported from Africa.

Abbreviations: F, female; M, male; NA, not applied; NS, not specified.

### 3.2. Etiology, Genetics, and Associated Anomalies

Polydactyly may occur in isolation or as part of syndromic conditions, with inheritance frequently autosomal dominant but also commonly sporadic [15]. Molecularly, limb duplication results from disruption of anteroposterior patterning pathways. In addition to HOX, SHH, BMP4, and apical ectodermal ridge signaling, recent genetic reviews emphasize the role of GLI3 and the ZRS regulatory enhancer of SHH, which function as antagonistic regulators of digit number and identity. GLI3 mutations are strongly associated with postaxial polydactyly, whereas alterations in the ZRS/SHH axis are more commonly linked to preaxial forms; overlapping phenotypes likely reflect shared developmental cascades [19, 20]. Mixed‐pattern polydactyly, such as in the present case, may therefore represent higher order disturbances within this integrated signaling network rather than single‐gene effects.

Preaxial polydactyly is particularly associated with additional congenital malformations, including syndactyly, vertebral anomalies, anorectal malformations, tibial deficiencies, cleft lip and palate, and cardiac defects, and may occur in syndromes such as Ellis–van Creveld, Rubinstein–Taybi, and Trisomy 13 [21, 22]. Consequently, comprehensive systemic evaluation and targeted imaging are recommended to exclude associated anomalies. Population‐based studies indicate that a meaningful proportion of neonates with polydactyly harbor additional anomalies, reinforcing the importance of cardiac and abdominal screening, particularly when preaxial elements are present.

In the current case, systemic evaluation was normal, and there was no family history of polydactyly, aligning with other reports of isolated heptadactylia and nonsyndromic lower extremity polydactyly [8, 9]. Although genetic testing was not performed due to the limited availability of molecular diagnostic services, the absence of syndromic features and normal systemic evaluation strongly supports an isolated sporadic developmental anomaly. However, lack of molecular confirmation limits definitive genotype–phenotype correlation, particularly involving pathways such as SHH–GLI3 signaling and ZRS enhancer regulation, which are known to influence digit patterning.

### 3.3. Classification and Anatomical Interpretation

Within the Temtamy–McKusick axis–based framework, this deformity is best interpreted as mixed preaxial–postaxial polydactyly, rather than central polydactyly or mirror foot [14, 15]. The medial component corresponds radiographically to first‐ray duplication at the metatarsal level, whereas the lateral component represents a fully developed postaxial (Type A) sixth ray, analogous to complete lateral ray polydactyly described in recent series [23, 24].

Importantly, there was no central ray duplication, no metatarsal bifurcation or shared bases, and no longitudinal epiphyseal bracket or delta phalanx, features commonly encountered in complex preaxial or central patterns [14, 24]. This anatomy supports a true combined border duplication rather than a variant of central polydactyly or mirror foot, which typically demonstrates medial ray repetition and, in some definitions, duplication of tarsal elements [12, 14, 25]. This distinction directly informs surgical planning.

### 3.4. Surgical Principles and Decision‐Making

Contemporary reviews emphasize that surgical goals in foot polydactyly are a plantigrade, stable, five‐ray foot with acceptable width that fits standard footwear, guided by careful radiographic and morphologic classification [24, 26, 27]. For lateral/postaxial polydactyly, multiple series highlight ray dominance, alignment, and metatarsal duplication as key determinants for choosing which ray to excise, with a trend toward excision of the nondominant or more deforming ray and recognition that residual metatarsal segments can maintain forefoot widening or joint deviation [23, 28, 29]. Central polydactyly and heptadactyly reports similarly prioritize resection of the most misaligned intercalary rays, reconstruction of the intermetatarsal ligament, and restoration of a narrow arch [8, 30].

**Figure 5:**
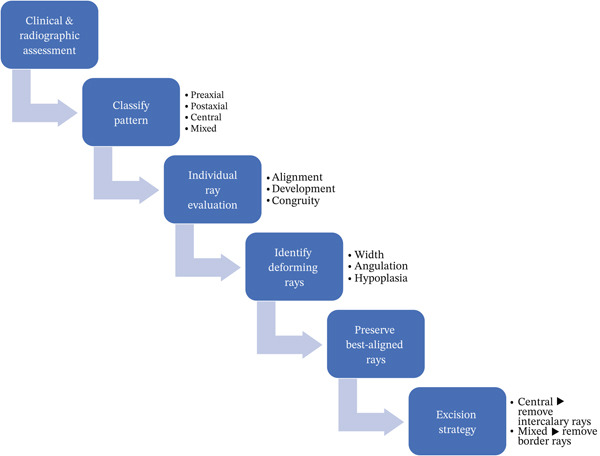
The algorithm for decision‐making for operative management.

Drawing on these principles, a practical algorithm (Figure 5) for mixed‐pattern heptadactyly includes: (1) individual assessment of each ray (development, alignment, and joint congruity); (2) identification of rays that most contribute to transverse widening or angular deformity; (3) preservation of the most aligned and structurally complete rays; and (4) excision of deforming rays typically border rays in combined preaxial–postaxial cases followed by selective soft‐tissue or ligament reconstruction only if instability or significant axis deviation remains [12, 28, 29].

In the present case, excision of the most medial and lateral rays addressed the primary deforming components, whereas the intermediate rays were structurally complete and well aligned. Stability was maintained without K‐wire fixation or ligament reconstruction, in contrast to central heptadactyly, where intermetatarsal ligament reconstruction is often required to maintain transverse arch narrowing [8, 30]. This alignment‐based, border‐ray strategy aligns with contemporary recommendations to avoid excessive reconstruction when transverse stability and joint congruity are preserved [15, 23, 24, 29].

### 3.5. Comparison With Central Heptadactyly

Central heptadactyly and central polydactyly present with duplication of middle rays and typically require excision of intercalary digits and reconstruction of soft tissues to restore the transverse arch while preserving medial and lateral borders [8, 12, 30]. In contrast, the current mixed preaxial–postaxial heptadactyly lacked central duplication; deformity and widening arose from medial and lateral border rays, necessitating border‐ray excision. Operative techniques described for mirror foot and complex preaxial patterns also rely on medial ray resection and arch reconstruction, but usually in the setting of more extensive tarsal involvement [12, 14, 25].

Thus, although both central and mixed forms are aimed at recreating a five‐ray foot of acceptable width, the surgical strategy is dictated by the axis of duplication: intercalary ray removal and ligament reconstruction in central heptadactyly, versus border‐ray excision with selective soft‐tissue procedures in mixed border duplications [7, 8, 31]. Accurate classification is therefore essential for appropriate planning and for meaningful comparison of outcomes across series.

### 3.6. Outcomes, Limitations, and Clinical Implications

Midterm studies across preaxial, central, and postaxial polydactyly report excellent functional scores and high rates of satisfactory radiographic alignment, although revision surgery is more common in complex preaxial patterns and in cases with residual metatarsal remnants or axis deviation [32]. Central polydactyly series show durable radiographic narrowing of the forefoot with near‐normal functional scores, but persistent subjective widening is common. A scoping review of lower extremity postaxial polydactyly found overall excellent long‐term function, with residual valgus and cosmetic concerns as the main issues; age at surgery had little effect on outcomes [31].

In this context, the early postoperative findings of a narrowed, symmetric forefoot and normal age‐appropriate limb use in the current case are consistent with literature documenting favorable early and midterm results after ray resection guided by detailed morphologic and radiographic assessment. Nonetheless, limitations must be explicitly acknowledged: absence of genetic testing and counseling, as well as lack of echocardiographic and abdominal ultrasound screening, limits definitive genotype–phenotype correlation or complete exclusion of subtle syndromic associations; follow‐up is short, so late growth‐related deformities, gait adaptations, or need for revision cannot be excluded; and standardized functional scoring or pedobarographic analysis, which have revealed persistent but clinically modest pressure alterations in other series, were not performed.

Overall, this case adds to the sparse literature on isolated mixed preaxial–postaxial heptadactyly and supports an individualized, alignment‐based border‐ray excision strategy that can achieve favorable early outcomes without routine fixation or ligament reconstruction when stability is preserved. Future reports with longer follow‐up, functional metrics, and genetic evaluation will be important to better define prognosis and refine surgical algorithms for this rare duplication pattern.

## 4. Conclusion

Heptadactyly of the foot is an extremely rare manifestation within the polydactyly spectrum, and presentations combining preaxial and postaxial duplication in a single foot are particularly uncommon. This case of a 9‐month‐old infant with unilateral heptadactyly and combined preaxial and postaxial polydactyly, without associated systemic anomalies, broadens the recognized phenotypic range of foot polydactyly.

Comprehensive clinical evaluation and targeted radiographic assessment are essential to confirm the anomaly as isolated and to guide the selection of rays for excision. Early elective surgical resection of supernumerary digits, with meticulous preservation of neurovascular structures and careful soft tissue reconstruction, can achieve a narrowed, functionally sound, and cosmetically acceptable foot, allowing attainment of developmental milestones without impairment.

Long‐term follow‐up into later childhood is important to monitor gait, footwear tolerance, and the development of any residual deformities. Documenting such rare configurations supports refinement of classification and surgical strategies for complex digital duplications and contributes valuable data to the sparse literature on heptadactyly of the foot.

## Author Contributions

Benardine Phillip Mallilah conceived the study, collected clinical data, performed the literature review, and drafted the manuscript. Philipo Godliving Moshi contributed to clinical management, critical revision of the manuscript, and supervision. Emmanuel Pastory Marua, Cuthbert Beita Kafanabo, and Getrude Laurent Mwasulile contributed to patient care, data interpretation, and manuscript review.

## Funding

No funding was received for this manuscript.

## Disclosure

All authors read and approved the final manuscript and agreed to be accountable for all aspects of the work.

## Ethics Statement

According to the St. Joseph Designated Hospital policy on case reports and the applicable Tanzanian national research ethics framework, ethical review board approval is not required for single‐patient case reports that involve anonymized clinical information collected during routine care and do not constitute human‐subject research. No experimental interventions were performed. Radiological and clinical images were fully anonymized, and all identifying patient information was removed from the manuscript and accompanying images before submission. The study was conducted in compliance with the Declaration of Helsinki principles for research involving human participants. Written informed consent for publication of clinical information and images was obtained from the patient′s parent/legal guardian.

## Consent

Written informed consent was obtained from the patient′s parent for publication of this case report and any accompanying images. A copy of the written consent is available for review by the editor‐in‐chief of this journal upon reasonable request.

## Conflicts of Interest

The authors declare no conflicts of interest.

## Supporting information


**Supporting Information** Additional supporting information can be found online in the Supporting Information section. 

## Data Availability

Data sharing is not applicable to this article as no datasets were generated or analyzed during the current study.
