# Supplementary material for: Isolated Unilateral Heptadactyly With Combined Preaxial and Postaxial Polydactyly of the Foot in a 9‐Month‐Old Infant
Source: Case Rep Orthop. 2026 Jun 18;2026:3435692. doi: 10.1155/cro/3435692 (PMC13278368; doi:10.1155/cro/3435692)
Supplement: Supplementary file 1 — Supporting Information Additional supporting information can be found online in the Supporting Information section. [file CRO-2026-3435692-s001.docx]

CARE Checklist Mapping Table

| CARE Item | Description | Location in Manuscript |
| --- | --- | --- |
| Title | Diagnosis/intervention identified as a case report | Title |
| Keywords | 2–5 keywords included | 5 Keywords section |
| Abstract | Background, case presentation, intervention, outcome, conclusion | Abstract |
| Introduction | Brief background and rationale | Introduction |
| Patient Information | Demographic characteristics, history, and family history | Case Presentation |
| Clinical Findings | Physical examination findings | Clinical Examination |
| Timeline | Chronological summary of events | Table 1 |
| Diagnostic Assessment | Radiological findings, classification, and diagnostic reasoning | Diagnostic Assessment |
| Therapeutic Intervention | Surgical procedure and perioperative management | Therapeutic Intervention |
| Follow-up and Outcomes | Clinical outcome and postoperative assessment | Follow-up and Outcomes |
| Discussion | Scientific discussion from relevant medical literature, rationale | Discussion |
| Patient Perspective | Parent/caregiver assessment of outcome | Follow-up and Outcomes |
| Informed Consent | Written informed consent obtained | Informed Consent Statement |
| Ethics | Institutional policy and ethics exemption described | Ethics Statement |
